# Comorbidities, acute kidney injury and long-term mortality in elderly patients hospitalized because of hip fracture: a moderation analysis

**DOI:** 10.1007/s40520-024-02771-1

**Published:** 2024-05-30

**Authors:** Saulo Lacerda Borges de Sá, Maria Luiza Medeiros Faria, Tiago Lins Oliveira Gonçalves, Alexandre Braga Libório

**Affiliations:** 1 Medical Sciences Postgraduate Program, Universidade de Fortaleza- UNIFOR, Fortaleza, Ceará Brazil; 2 Medical Course, Universidade de Fortaleza-UNIFOR, Fortaleza, Ceará Brazil

**Keywords:** Acute kidney injury, Hip fracture, Mortality

## Abstract

**Introduction:**

Femoral fractures in elderly individuals present significant health challenges, often leading to increased morbidity and mortality. Acute kidney injury (AKI) during hospitalization further complicates outcomes, yet the interaction between AKI severity and comorbidities, as quantified by the Charlson Comorbidity Index (CCI), remains poorly understood in this population. This study aimed to assess the associations between AKI severity and the CCI and between AKI severity and one-year mortality postfemoral fracture in elderly patients.

**Methodology:**

This study utilized data from the Multiparameter Intelligent Monitoring in Intensive Care (MIMIC-IV) database and focused on elderly patients (> 65 years) admitted with hip fractures. Patients were categorized based on AKI stage according to the KDIGO criteria and CCI scores. The primary outcome assessed was all-cause mortality one year after hospital discharge. The statistical analyses included logistic regression, Cox proportional hazards regression and moderation analysis with the Johnson–Neyman technique to evaluate associations between AKI and long-term mortality and between the CCI and long-term mortality.

**Results:**

The analysis included 1,955 patients and revealed that severe AKI (stages 2 and 3) was independently associated with increased one-year mortality. Notably, the CCI moderated these associations significantly. A lower CCI score was significantly correlated with greater mortality in patients with severe AKI. The impact of severe AKI was greater for those with a CCI as low as 3, more than doubling the observed one-year mortality rate. In contrast, higher CCI scores (≥8) did not significantly impact mortality. Sensitivity analyses supported these findings, underscoring the robustness of the observed associations.

**Conclusion:**

This study elucidates the complex interplay between AKI severity and comorbidities and long-term mortality in elderly hip fracture patients. These findings underscore the importance of considering both AKI severity and comorbidity burden in prognostic assessments and intervention strategies for this vulnerable population. Targeted interventions tailored to individual risk profiles may help mitigate the impact of AKI on mortality outcomes, ultimately improving patient care and outcomes. Further research is warranted to explore the underlying mechanisms involved and refine risk stratification approaches in this population.

**Supplementary Information:**

The online version contains supplementary material available at 10.1007/s40520-024-02771-1.

## Introduction

Femoral fractures present a significant health challenge for the elderly population and lead to substantial morbidity, disability, mortality, and economic burdens on healthcare systems and caregivers worldwide [1]. With the ongoing demographic shift toward an aging population, the incidence of hip fractures is expected to increase. By the year 2050, the annual global incidence of hip fractures is projected to exceed six million [2]. While mortality rates may vary depending on population demographics and the specific anatomical location of the fracture, one-year follow-up typically reveals rates ranging from 20 to 25% [3, 4].

Acute kidney injury (AKI) is a common complication during hospitalization for hip fractures and has an incidence rate of 24% [5, 6]. In addition to prolonging hospital stays and increasing in-hospital mortality, AKI has been associated with elevated long-term mortality [7]. Several studies have investigated the effects of AKI on long-term mortality after hip fracture [8–11]. For example, Hong et al. reported a hazard ratio (HR) of 1.63 after a median follow-up exceeding three years, and other studies reported an HR of 2.72 after one year of follow-up [8, 11]. 

Given the age-related increase in hip fracture incidence, the comorbidity incidence in this population is high, affecting both short- and long-term prognoses. The Charlson Comorbidity Index (CCI), a system designed to quantify morbidity, serves as a crucial tool for prognostic assessment [12]. Previous research has underscored the significance of the CCI in predicting the prognosis of hip fracture patients [13, 14].

Although accumulating evidence suggests that AKI is associated with long-term sequelae (such as chronic kidney disease, cardiovascular events, and mortality) in the general population [7, 15], studies evaluating the impact of AKI in patients with a high prevalence of comorbidities and whether these comorbidities can modify such associations are rare. Yalin et al. evaluated the relationship between the CCI score and AKI-related hospital mortality, but no discernible associations were found [16].

This study aimed to evaluate long-term mortality in elderly patients following hospitalization for hip fractures based on AKI grade and the association of AKI grade with mortality according to the CCI. We hypothesized that the CCI can modulate the association between AKI and long-term mortality.

## Methods

### Multiparameter intelligent monitoring in the intensive care II database and data collection

The Multiparameter Intelligent Monitoring in Intensive Care (MIMIC-IV) project is managed by the Massachusetts Institute of Technology Laboratory for Computational Physiology and houses data on patients admitted to Beth Israel Deaconess Medical Center from 2008 to 2019 [17, 18]. The database is publicly available, and researchers who agree to the data use agreement and have completed “protecting human subjects training” can request access. This study received approval from the institutional review boards of the Massachusetts Institute of Technology and Beth Israel Deaconess Medical Center and was granted a waiver of informed consent.

We included all elderly patients (aged > 65 years) whose primary reason for hospital admission was hip fracture; patients were classified according to the International Classification of Diseases (ICD) versions 9 and 10; and patients who were discharged alive. The discharge summary of those with nonspecified types of femur fractures according to the ICD was revised. Patients without at least two serum creatinine (sCr) measurements within a 7-day interval, those with baseline sCr > 4 mg/dL, those with known chronic kidney disease (CKD) stage 5 (creatinine clearance < 15 mL/min/1.^73 m2^), and those undergoing maintenance hemodialysis before hospital admission were excluded. Patients with missing values were excluded. Patients with a hospital length of stay (LOS) < 24 h were excluded.

### Demographic and clinical variables

Patient demographic characteristics (age and sex), comorbidities, femur fracture type, surgical procedure, nephrotoxic medications, and AKI classification according to the Kidney Disease: Improving Global Outcomes (KDIGO) criteria were collected. The following types of hip fractures were classified according to anatomic site: femoral neck, transtrochanteric and subtrochanteric. Patients were categorized based on their sCr levels, and those transferred to the intensive care unit (ICU) were also categorized based on their urine output during their ICU stay. The comorbidities recorded in ICD versions 9 and 10 were collected and included chronic kidney disease, myocardial infarction, congestive heart failure, liver disease, chronic obstructive pulmonary disease, hypertension, diabetes mellitus, dementia, and cancer. The baseline estimated glomerular filtration rate (eGFR) was calculated using the CKD Epidemiology Collaboration (CKD-EPI) equation − 2021 with the lowest sCr during the hospital stay. Finally, the CCI was calculated accordingly.

### Outcome

Patients who were discharged alive were stratified by KDIGO stage, and the outcome was all-cause mortality one year after hospital discharge.

### Statistical analysis

The descriptive characteristics of the study population are presented as proportions for categorical and binary variables, means with standard deviations (SDs) for normally distributed continuous variables, and medians with interquartile ranges (IQRs) for skewed variables. Assumptions of normality were validated by a quantile–quantile plot and histogram. Differences between categorical variables were assessed using chi-square tests and t tests or Wilcoxon signed-rank tests, as appropriate.

Cox proportional hazards regression models were used to assess the association between AKI stage and long-term mortality. The models, including moderation analysis, were adjusted for demographic factors (age, sex), CCI score, type of hip fracture, need for surgical intervention and baseline eGFR. Before Cox regression was performed, we tested the linearity of continuous variables with outcome and verified the absence of collinearity.

For the moderation analysis, the interaction model of logistic regression was employed, with additional product interaction terms of AKI stage and CCI. The Johnson–Neyman technique [19] was used to identify significant transition points in the moderation model. This technique analyses the significance between predictor and outcome variables by continuously aligning the moderating variable (CCI) and computing regions of significance for interactions. This analysis was performed only after the interaction was significant. The moderator level was then defined as the threshold detected by the Johnson–Neyman technique, where the significance between the predictor and outcome was observed. All models were adjusted for the aforementioned covariates, and a p value < 0.05 was used to indicate statistical significance. All analyses were conducted using SPSS (version 20.0; IBM, Armonk, NY, USA).

## Results

### Patient characteristics

**Fig. 1:**
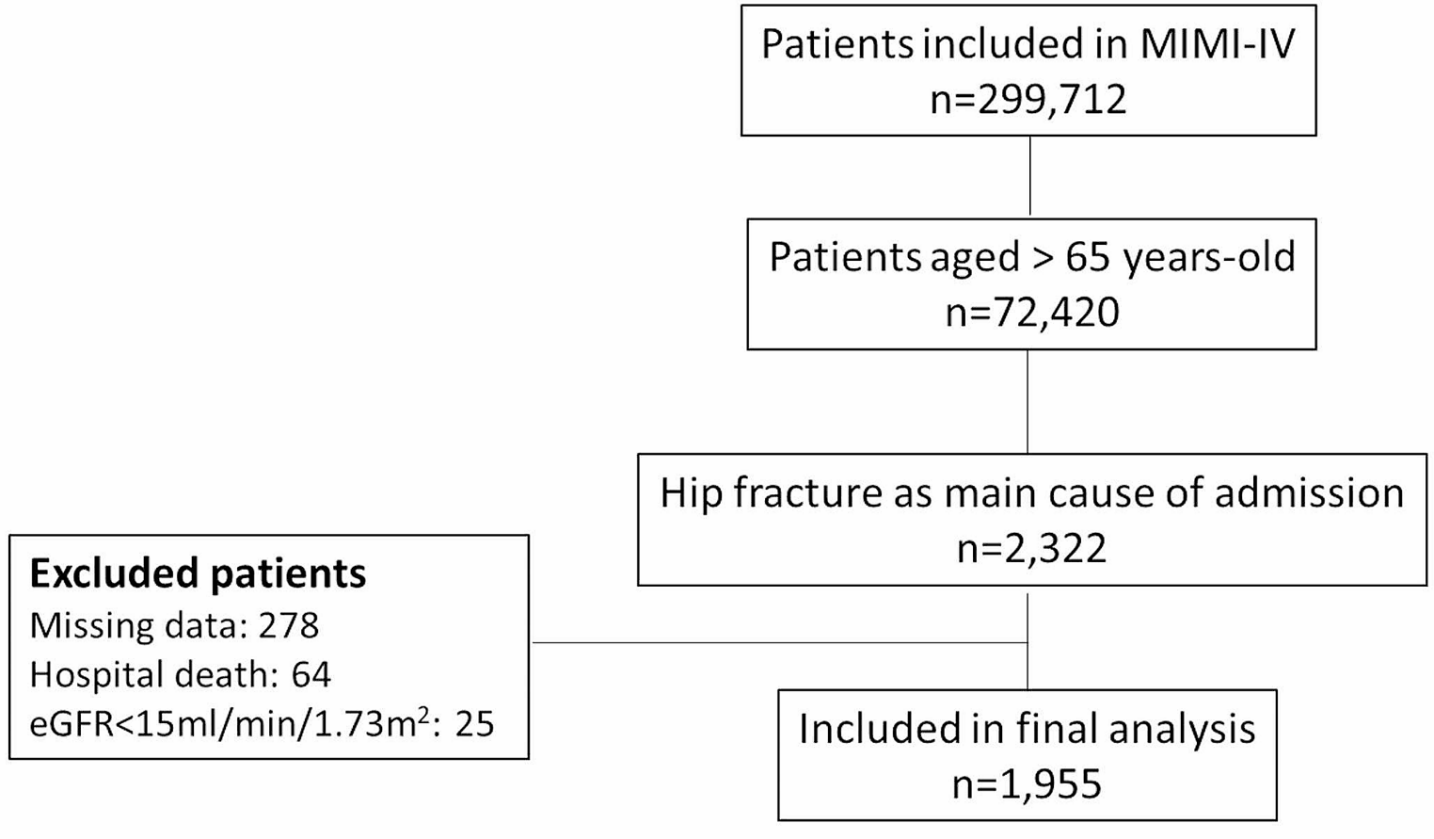
Flowchart of the included patients from the MIMIC-IV database

The MIMIC-IV database contains the records of 299,712 patients, 72,420 of whom were older than 65 years at the time of admission. Among these patients, 2,322 had fractures as the main reason for hospital admission. Because of missing data, 278 patients were excluded. Additionally, 25 patients were excluded because their eGFR was < 15 mL/min/m^2,^ and 64 patients were excluded because of hospital death. Overall, 1,955 patients remained in the final analysis (see Fig. 1).

The mean age was 81.9 ± 7.8 years, and 1,400 (71.6%) patients were female. The majority of patients had neck and transtrochanteric femur fractures (*n* = 1,797, 91.9%). During the hospital stay, 194 (9.9%) patients were admitted to the ICU, for a median length of stay of 1.73 [0.99–3.01] days. The median length of hospital stay was 4.7 (3.7–6.5) days. The median CCI was 5 (4–7). Almost half of the patients (*n* = 972, 49.7%] developed AKI; a complete description can be found in Table 1.

**Table 1:** Characteristics of femur fracture patients according to survival status within one year

	All patients (*n* = 1,955)	Alive (*n* = 1,505)	Died (*n* = 450)	*p*
Age (years), mean±SD	81.9 ± 7.8	81.1 ± 7.9	84.4 ± 7.1	< 0.001
Female, *n* (%)	1,400 (71.6)	1,11 (73.8)	289 (64.2)	< 0.001
*Comorbidities* Hypertension, n(%) Diabetes Mellitus, n(%) Congestive heart failure, *n* (%)	1,293 (66.1) 428 (21.9) 436 (22.3)	1,034 (68.7) 322 (21.4) 278 (18.5)	259 (57.6) 106 (23.6) 158 (35.1)	< 0.001 0.33 < 0.001
Charlson comorbidity index, median [IQR}	5 [4–7]	5 [4–7]	7 [5–9]	< 0.001
*Type of femur fracture* Neck, n(%) Transtrochanteric, n(%) Subtrochanteric, n (%)	874 (44.7) 923 (47.2) 158 (8.1)	663 (44.1) 709 (47.1) 133 (8.8)	211 (46.9) 214 (47.6) 25 (5.5)	0.14
Surgical procedure, n (%)	1,273 (65.1)	999 (66.4)	274 (60.9)	0.032
ICU admission, n (%)	194 (9.9)	127 (8.4)	67 (14.9)	< 0.001
eGFR (ml/min/m^2^), mean ± SD	77.3 ± 21.4	78.7 ± 20.5	72.6 ± 23.5	< 0.001
AKI stage No-AKI, n (%) Stage 1, n (%) Stage 2, n (%) Stage 3, n (%)	983 (50.3) 701 (35.9) 214 (10.9) 57 (2.9)	813 (54.0) 515 (34.2) 141 (9.4) 36 (2.4)	170 (37.8) 186 (41.3) 73 (16.2) 21 (4.7)	< 0.001
Length of hospital stay (days), median [IQR]	4.7 [3.7–6.5]	4.6 [3.7-6.0]	5.3 [ 4.0-7.7]	< 0.001

### Variables associated with one-year mortality

**Fig. 2:**
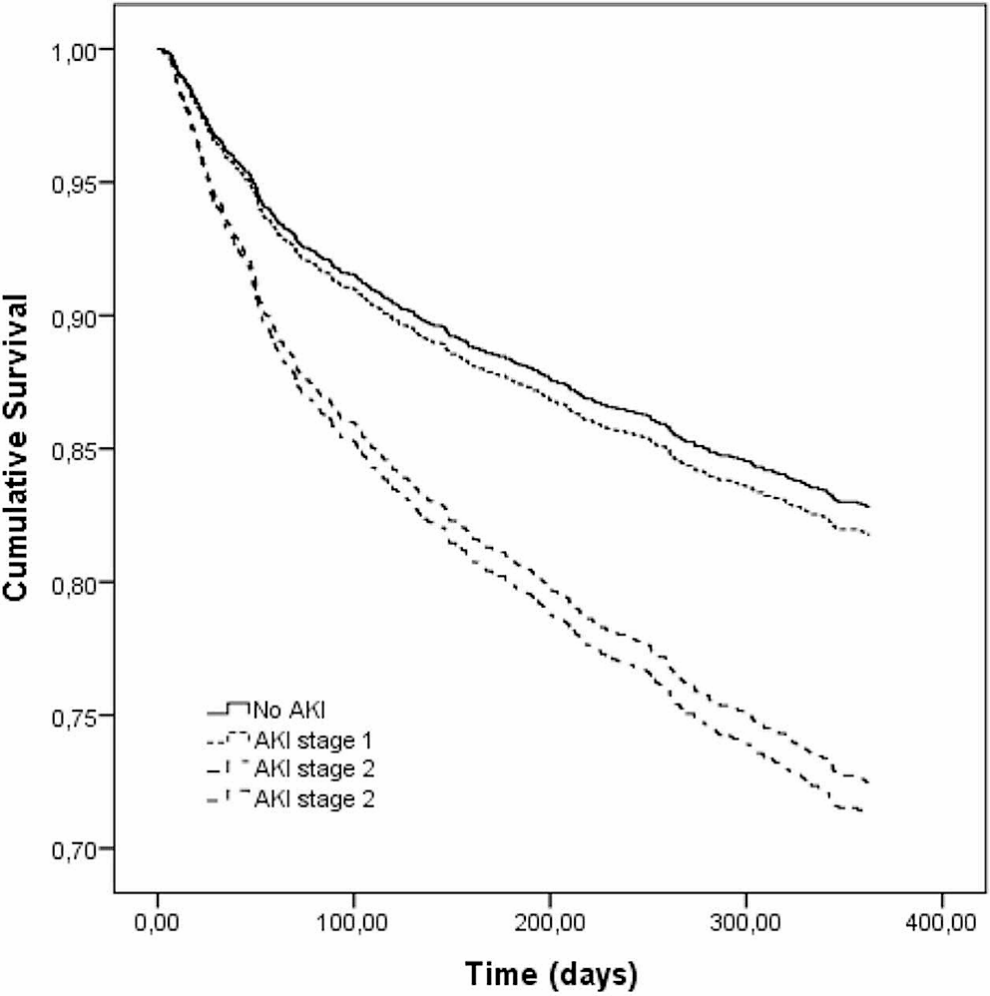
Cox survival curves for mortality according to AKI stage. Significant difference was observed between patients with no-AKI/stage 1 AKI and patients with AKI stage 2 or 3

The univariate differences between survivors and nonsurvivors after one year of discharge are shown in Table 1. Generally, patients who died were older and had a greater CCI, more congestive heart failure and fewer hypertension diagnoses. Additionally, fewer patients underwent femur-related surgical procedures, had more passages through the ICU, had a longer length of hospital stay and developed more AKI in all stages than did the surviving patients. According to the Cox proportional hazards model, age, male sex, no surgical intervention, CCI score and AKI stage were independently related to one-year survival (Table 2). Figure 2 displays Cox survival curves according to AKI stage. A significant difference was observed only between patients with no AKI or AKI stage 1 and patients with severe AKI (stages 2 or 3).

**Table 2:** Variables associated with increased risk of one-year mortality after adjustment for age, gender, Charlson Comorbidity Index, type of femur fracture, need for surgical intervention and baseline eGFR

Variable	Hazard Ratio	95.0% CI for Hazard Ratio
AKI stage AKI stage 1 AKI stage 2 AKI stage 3	1.098 1.709 1.901	0.899–1.325 1.365–2.370 1.254–2.704
Male gender	1.278	1.044–1.564
Age (years)	1.063	1.048–1.078
No-surgical intervention	1.258	1.038–1.524
CCI	1.277	1.233–1.323

### The charlson comorbidity index moderates the association between AKI and long-term mortality

**Fig. 3:**
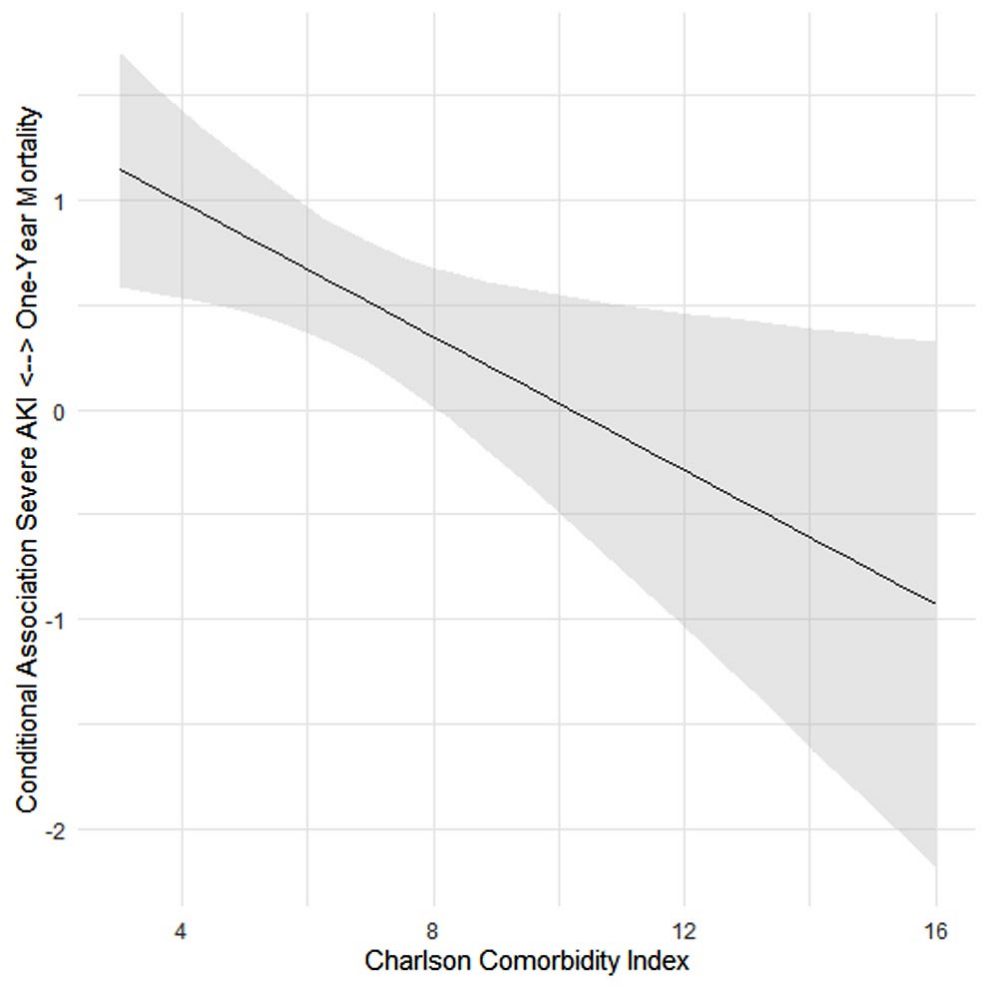
Moderation effects of the Charlson Comorbidity Index on the conditional association between severe AKI and one-year mortality. Note that the lower 95% confidence interval crosses the zero value (no association) at values near 8, and up to this value, there is no significant association

**Fig. 4:**
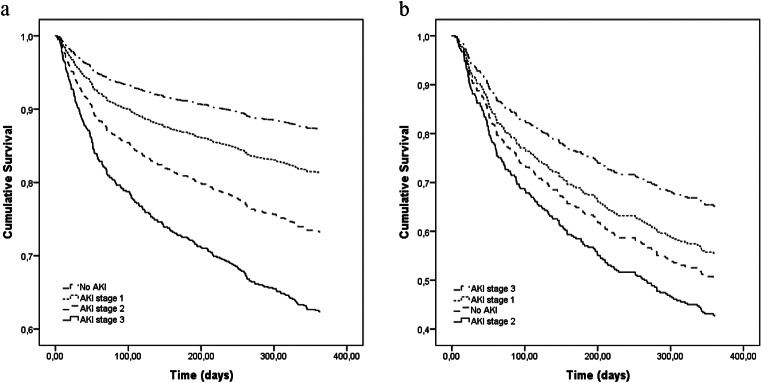
Cox survival curves for one-year mortality in patients according to the Charlson comorbidity index (CCI). A significant difference was observed in the subgroup with a CCI < 8 according to AKI stage (Fig. 4a). In the subgroup with a CCI ≥ 8, there was no significant difference between patients according to AKI stage. Note that there is no AKI stage order in Fig. 4b

Because only AKI stages 2 and 3 were independently associated with one-year mortality, we conducted moderated regression analyses to assess whether there was an interaction effect between the CCI and severe AKI (stages 2 and 3). We identified a significant moderation effect for the relationships between the CCI and severe AKI and mortality (β coefficient = -0.161, *p* = 0.003). Figure 3 shows the moderating effects of the CCI on the conditional association between severe AKI and one-year mortality. The conditional association was significant only when the CCI reached 8. In patients with a CCI greater than 8, severe AKI had no significant effect on one-year mortality. The conditional association was greatest in patients with a CCI up to 3, with a β coefficient value of 1.15. This finding indicated that severe AKI was associated with more than double the one-year mortality in patients with such a CCI. In contrast, patients with a CCI greater than 8 and severe AKI during the hospital stay had no significant impact on one-year mortality. To further illustrate the moderation effect of the CCI on the association between severe AKI and one-year mortality, we plotted two survival curves (with a low or high CCI and a CCI of 8 as the cutoff point) in which the patients were divided according to AKI stage. We observed that in patients with a higher CCI, the curves overlapped, with no association between AKI stage and mortality. In patients with a low CCI, there was a step reduction in survival according to AKI stage (Fig. 4a and b).

We also tested each comorbidity included in the CCI individually. The β coefficients are shown in supplementary Table S1. There was a significant interaction effect only for stroke and severe AKI when one-year mortality was the outcome.

### Sensitivity analysis

We performed two sensitivity analyses. The first included only very older patients (> 75 years old) (*n* = 1,554). Another sensitivity analysis was performed only for patients who presented with neck femur fractures (known as intracapsular fractures) (*n* = 874). The results were very similar, with significant interactions between the CCI and severe AKI (β coefficient = -0.168, *p* = 0.03 and β coefficient = -0.233, *p* = 0.01, respectively). The conditional associations according to the CCI are shown in tabllementary figures S1 and S2 for both sensitivity analyses.

## Discussion

In this study, we evaluated a large retrospective cohort of older patients admitted for hip fracture and with data about mortality one year after hospital discharge. Our main findings included first determining the main variables associated with one-year mortality, including severe AKI (stages 2 and 3) and the CCI. We also further explored the association between severe AKI and one-year mortality and revealed that the CCI, beyond being a strong predictor of one-year mortality, is an important moderator of this association. Our data revealed that patients with reduced comorbidities (CCI < 8) and, consequently, a longer life expectancy are more impacted by long-term outcomes due to severe AKI. In contrast, AKI had no association with one-year mortality in patients with a high CCI.

In the present study, our mortality rate was similar to that of other studies evaluating hip fracture [20, 21]. We also confirmed some findings of previous studies about long-term mortality in hip fracture patients: an independent association between age, comorbidities and AKI [20]. Additionally, we demonstrated that severe AKI (stage 2 or 3) was independently associated with one-year mortality. Many studies have demonstrated that AKI has an impact on long-term mortality in the general population [7, 15, 22]. Additionally, several studies have evaluated the risk factors for AKI after hip fracture [23–26] and its impact on long-term mortality [8–11]. In our study, only severe AKI (stage 2 and 3) was associated with one-year mortality. We were unable to observe a significant association between stage 1 AKI and one-year mortality.

Overall, our study demonstrated that the CCI is strongly associated with one-year mortality. Moreover, the CCI has an important moderating effect on the association between severe AKI and one-year mortality. In fact, severe AKI had no impact on the mortality of patients with a CCI≥8 but more than doubled the mortality in those with a CCI near 3. Several mechanisms may underlie the observed moderation effect. Patients with a higher comorbidity burden may already be at a higher baseline risk of mortality due to their underlying health conditions, and the impact of AKI is attenuated. This hypothesis is supported by the findings of one study in which a CCI greater than 7 was associated with 5-year mortality (odds ratio of 13.6) compared with a CCI < 7 in patients with femur fracture [27]. Additionally, we suggest that the presence of multiple comorbidities may complicate the management of AKI, leading to suboptimal treatment and poorer outcomes. A large observational study demonstrated that the use of angiotensin-converting enzyme inhibitors (ACEIs) or angiotensin receptor blockers (ARBs) after hospital discharge is associated with better outcomes in patients with AKI [28]. However, the use of these medications in patients with greater comorbidity burdens is cumbersome.

The findings of our study have important clinical implications. First, they highlighted the need for risk stratification in femoral fracture patients to identify those at higher risk of mortality. Patients with a lower burden of comorbidities who develop AKI may benefit from closer monitoring and early intervention to mitigate the impact of AKI on mortality. Conversely, in patients with a greater comorbidity burden, efforts should likely focus on comprehensive management of comorbid conditions to reduce overall mortality risk, even in the absence of a significant association between AKI and mortality. Although we evaluated only mortality, it is also important to consider the potential impact of AKI on other long-term outcomes. AKI has been associated with an increased risk of chronic kidney disease [29], cardiovascular events [30], and reduced quality of life in survivors [31]. Therefore, efforts to prevent and manage AKI in femoral fracture patients should extend beyond acute care to include strategies for long-term follow-up and surveillance.

Our study has several important limitations. First, our study has a retrospective design, which precludes causal inference. Second, the inclusion criteria were based on ICD codes, which may be subject to misclassification bias. To reduce bias at hospital admission due to other medical conditions, we included only patients with hip fracture as the main admission diagnosis. Third, we did not have data after hospital discharge. It is likely that functional capacity after hospital stay and medication, as stated above, have an impact on mortality. Additionally, the generalizability of our findings may be limited to similar patient populations in similar healthcare settings. However, future research should explore the underlying mechanisms driving the moderation effect of comorbidities on the association between AKI and mortality to evaluate whether this association persists in the general population.

In conclusion, our study provides evidence that the CCI moderates the association between acute kidney injury and one-year mortality in hospitalized femoral fracture patients. Although AKI independently predicts increased mortality in patients with fewer comorbidities, this association is not evident in patients with a greater comorbidity burden. These findings underscore the importance of considering comorbidity burden when assessing the impact of AKI on mortality outcomes and have implications for the clinical management of femoral fracture patients. Further research is warranted to elucidate the underlying mechanisms driving this moderation effect and to develop targeted interventions aimed at improving outcomes in high-risk patients.

### Electronic supplementary material

Below is the link to the electronic supplementary material.

Supplementary Material 1

Supplementary Material 2

Supplementary Material 3

## Data Availability

No datasets were generated or analysed during the current study.
