# Supplementary material for: Comorbidities, acute kidney injury and long-term mortality in elderly patients hospitalized because of hip fracture: a moderation analysis
Source: Aging Clin Exp Res. 2024 May 30;36(1):123. doi: 10.1007/s40520-024-02771-1 (PMC11136753; doi:10.1007/s40520-024-02771-1)

**Supplementary figure 1:** Moderation effects of the Charlson Comorbidity Index on the conditional association between severe AKI and one-year mortality in very older patients aged>75 years (n=1,554). Note that the lower 95% confidence interval crosses the zero value (no association) at values near 8, and up to this value, there is no significant association.


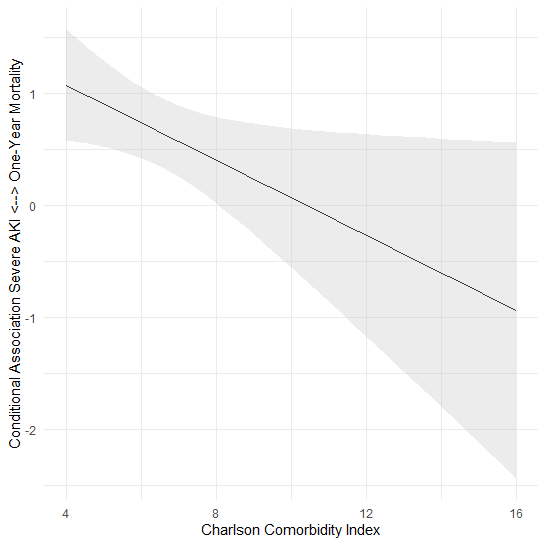

Supplement: Supplementary file 1 — Supplementary Material 1 [file 40520_2024_2771_MOESM1_ESM.docx]
