# Supplementary material for: Comorbidities, acute kidney injury and long-term mortality in elderly patients hospitalized because of hip fracture: a moderation analysis
Source: Aging Clin Exp Res. 2024 May 30;36(1):123. doi: 10.1007/s40520-024-02771-1 (PMC11136753; doi:10.1007/s40520-024-02771-1)
Supplement: Supplementary file 3 — Supplementary Material 3 [file 40520_2024_2771_MOESM3_ESM.docx]

| **Variable** | **Beta Coefficient** | **95% CI** |
| --- | --- | --- |
| Age | 0.24 | -0.14 to 0.62 |
| Myocardial infarction | -0.53 | -.132 to 0.21 |
| Congestive heart failure | -0.46 | -0.97 to 0.07 |
| Peripheral vascular disease | -0.34 | -0.20 to 1.55 |
| Cerebrovascular disease | -1.53 | -2.63 to -0.49 |
| Dementia | 0.19 | -0.51 to 0.90 |
| Chronic pulmonary disease | -0.48 | -1.02 to 0.09 |
| Connective tissue disease | 0.45 | -0.75 to 1.62 |
| Ulcer disease | -1.14 | -3.80 to 1.54 |
| Diabetes | -0.45 | -0.99 to 0.16 |
| Paraplegia | -1.62 | -4.20 to 0.96 |
| Renal disease | -0.38 | -0.99 to 0.22 |
| Liver disease | -0.35 | -1.31 to 1.99 |
| Metastatic solid tumor | 0.35 | -1.35 to 2.01 |
| AIDS | - | - |

**Supplementary Table S1:** Interaction terms and 95% CIs for all Charlson Comorbidity Indices with severe AKI to predict one-year mortality. According to our data, there were no patients diagnosed with AIDS. The interaction term was significant only for cerebrovascular disease
